# *Ab initio* phasing of the diffraction of crystals with translational disorder

**DOI:** 10.1107/S2053273318015395

**Published:** 2019-01-01

**Authors:** Andrew J. Morgan, Kartik Ayyer, Anton Barty, Joe P. J. Chen, Tomas Ekeberg, Dominik Oberthuer, Thomas A. White, Oleksandr Yefanov, Henry N. Chapman

**Affiliations:** a Center for Free-Electron Laser Science, Deutsches Elektronen-Synchrotron DESY, Notkestrasse 85, 22607 Hamburg, Germany; b Department of Physics, Arizona State University, Tempe, AZ, 85287, USA; c Department of Physics, University of Hamburg, Luruper Chaussee 149, 22761 Hamburg, Germany; d Centre for Ultrafast Imaging, Luruper Chaussee 149, 22761 Hamburg, Germany

**Keywords:** X-ray diffraction, diffuse scattering, phase retrieval, macromolecular crystallography

## Abstract

To date X-ray protein crystallography is the most successful technique available for the determination of high-resolution 3D structures of biological molecules and their complexes. In X-ray protein crystallography the structure of a protein is refined against the set of observed Bragg reflections from a protein crystal. The resolution of the refined protein structure is limited by the highest angle at which Bragg reflections can be observed. In addition, the Bragg reflections alone are typically insufficient (by a factor of two) to determine the structure *ab initio*, and so prior information is required. Crystals formed from an imperfect packing of the protein molecules may also exhibit continuous diffraction between and beyond these Bragg reflections. When this is due to random displacements of the molecules from each crystal lattice site, the continuous diffraction provides the necessary information to determine the protein structure without prior knowledge, to a resolution that is not limited by the angular extent of the observed Bragg reflections but instead by that of the diffraction as a whole. This article presents an iterative projection algorithm that simultaneously uses the continuous diffraction as well as the Bragg reflections for the determination of protein structures. The viability of this method is demonstrated on simulated crystal diffraction.

## Abstract 2

This article reports on the combined use of Bragg reflections and diffuse scatter for structure determination in crystallography.

## 1. Introduction   

The diffraction of coherent radiation from an object onto a detector placed far from the object gives rise to smoothly varying diffraction features that are bandwidth limited by the size of the object. The detector measures the intensity, the mean-squared value of the electric field amplitude, but not the phases of the scattered radiation. If the phases were known, then one could synthesize an image of the object directly by numerical propagation of the wavefront of the coherent field from the detector back to the sample. This image would be proportional to the electron density or scattering strength of the object. However, without the phases, the numerical transformation of the measured intensities only yields a map of the pair correlations, also known as the autocorrelation of the object density, of the point scatterers in the object.

Despite the missing phase information, it is often the case that an image of a single object of finite extent can be reconstructed from the diffraction intensities without prior knowledge if those smoothly varying diffraction intensities of the object are sufficiently sampled according to the Nyquist–Shannon sampling criterion (Nyquist, 2002 ▸; Shannon, 1949 ▸; Bates & McDonnell, 1986 ▸). Such a reconstruction can be achieved using a class of iterative projection algorithms (IPAs) to solve for the missing phases where only the intensities have been measured (Marchesini *et al.*, 2003 ▸; Dronyak *et al.*, 2009 ▸; Marchesini, 2007 ▸). If many copies of the object are packed into a periodic array, for example in a crystal, then the diffraction intensities are greatly enhanced at specific scattering angles corresponding to Bragg reflection angles. The enhancement factor is equal to the number of repeating objects, which even in a small macromolecular crystal is large enough to make such diffraction measurable. The diffraction pattern in this case consists of Bragg peaks which have a width that is inversely proportional to the side-length of the crystal and which are, in general, spaced at intervals that are not fine enough to satisfy the Nyquist–Shannon criterion of the unit-cell contents. Thus the Bragg reflections of a crystal are said to ‘under-sample’ the molecular diffraction of the unit cell. This is the well known ‘phase problem’ and the reason that the phases of the Bragg peaks cannot be readily determined from the diffraction alone. It is the central problem that every crystallographic phasing method must overcome.

We see therefore that the phase problem in crystallography stems from the under-sampled diffraction intensities by the Bragg reflections and can more rightly be considered as an intensity problem (Thibault & Elser, 2010 ▸); many experimental and computational strategies have been employed to increase the measurable information from macromolecular crystals in order to solve the structure. These methods either require specific properties of the sample, such as the presence of heavy atoms, or partial chemical models to gain this information – see for example Rupp (2009 ▸) for a description of these methods in the present context. They also depend upon the measurement of high-resolution diffraction to ensure a large number of measurements compared with fitting parameters in the model. Obtaining well diffracting crystals to give the necessary high resolution is one of the largest bottlenecks in the structure determination pipeline. Macromolecules in crystals are usually only tenuously connected to each other, leaving large voids throughout the crystal that are filled with solvent. The fraction of the volume of this solvent often can exceed 50% (Chruszcz *et al.*, 2008 ▸), in which case the Bragg reflections actually do over-sample the molecular transform (even though they under-sample the unit-cell diffraction). For this condition, it becomes possible to apply IPAs to directly phase the diffraction without any need of a model, high-resolution data or specific structural characteristics (Millane & Stroud, 1997 ▸; Lo *et al.*, 2016 ▸; He & Su, 2015 ▸), although Liu *et al*. suggest that a solvent fraction of at least 65% is required in practice (Liu *et al.*, 2012 ▸).

More recently, it was found that translational disorder in crystals of the membrane protein complex photosystem II (PSII) gives rise to continuous diffraction that can be phased using an IPA (Ayyer *et al.*, 2016 ▸). Random and independent displacements of rigid units (the PSII dimer) from lattice sites disrupt the formation of Bragg peaks at high resolutions, and instead give rise to the incoherent sum of the single-molecule (continuous) diffraction from the rigid objects. This presents an opportunity to greatly increase the information content of the measured diffraction to allow direct imaging (that is, *ab initio* phasing), but also raises a challenge in how to best utilize both the Bragg and continuous diffraction. In our previous work (Ayyer *et al.*, 2016 ▸) these two types of diffraction were treated separately, with the continuous diffraction used to extend the resolution of a map that was initially refined from the Bragg data. Here we present an IPA that uses both types of diffraction on equal footing to recover an image of the rigid object in a translationally disordered crystal. The method generalizes iterative phasing of crystal diffraction data and combines ideas from the field of coherent diffractive imaging with analysis concepts such as those used in molecular replacement. We require that the contribution to the diffuse scatter from other types of disorder in the crystal (except for uncorrelated random atomic displacements and solvent disorder) is absent, or at least insignificant, compared with the uncorrelated rigid-body translations of the molecule/s. It should also be noted that we do not provide, nor are we aware of, any definitive prior test for establishing when these conditions are satisfied.^1^

## 2. Diffraction model of the crystal   

We consider the mathematical description of a crystal that is generated from a single rigid unit [with density 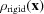 at position 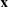]. This rigid unit may be what is generally thought of as the asymmetric unit of the crystal, or it may be a particular molecular complex. We consider for now that there is only one repeating rigid unit, but more generally there could be several types, such as two domains of a molecule. The unit-cell density can be generated from the single rigid unit along with the crystal symmetry and the unit-cell dimensions via rotation and translation operations 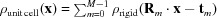, where the sum is over the *M* symmetry-related copies of 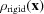 in the unit cell. Here 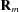 is the rotation matrix for the *m*th copy and 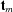 is the translation vector.

In a perfect crystal without any translational disorder, each rigid unit of each unit cell within the crystal is located at the ideal lattice sites 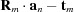, where 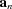 are the lattice points that define the entire crystal consisting of *N* unit cells. In a crystal with translational disorder each rigid unit (*m*) of each unit cell (*n*) is displaced from its ideal location by an amount 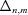. We consider displacements drawn from a normal distribution such that 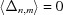 and 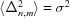. The crystal density 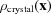 can be generated by a convolution of the disordered lattice of *N* points with each of the *M* rigid units:
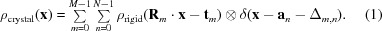
It can be shown, for example see Ayyer *et al.* (2016 ▸), that the diffraction intensities of such a crystal are given by
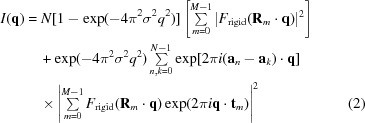
where 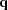 (reciprocal to 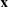) is given in terms of the wavelength λ and the angle between the incoming and outgoing rays θ such that 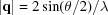, 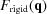 is the molecular transform of the rigid unit [equal to the Fourier transform of 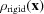] and we assume that the crystal is coherently illuminated. Measurements of the intensity are made at samples 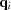 (for pixel *i*) by a pixellated detector placed far from the crystal.

The second term in equation (2) is the usual formulation for the Bragg peak intensities, formed by the square of the coherent sum of the scattering from each of the *M* rigid units in their respective mean positions and orientations in the unit cell. These Bragg peak intensities are modulated by the Debye–Waller factor 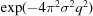, which decreases from 1 to 0 as the scattering angle increases. The Debye–Waller factor arises because the Bragg peaks only give information about the average structure of the unit cell. In this case the average structure is blurry in real space due to the random displacements, and the effect of this blurring is to diminish the strength of the Bragg peaks at high resolution (or scattering angle) according to this factor. The first term of equation (2) is the *incoherent* sum of the square modulus of the scattered light from each of the rigid units. The incoherent sum is similar to twinning in crystallography, except that here the sum is over the possible orientations of the rigid unit rather than the possible orientations of the crystal. It is modulated by the complementary Debye–Waller factor, which increases from 0 to 1 with increasing scattering angle. We note that distributions of the translations 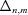 could be considered other than Gaussian, in which case the factors multiplying the two terms in equation (2) take on different forms as given by the correlation of 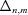. Other forms of disorder may additionally occur in the crystal, for example random and independent displacements of atoms in all molecules that will give rise to another Debye–Waller factor that modulates the entire diffraction pattern.

At first glance it may appear that the ratio of the continuous to the Bragg peak diffraction intensities [arising from the first and second terms in equation (2), respectively] scales with the number of unit cells in the crystal. However it is σ (rather than *N*) that determines the relative strength of the diffuse scatter to the Bragg reflections in each resolution shell and as a whole. Although the Bragg peak heights scale as 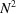, the solid angle is inversely related to crystal size, giving a signal of integrated counts that scales as *N*. In today’s detectors, the width of the Bragg peak will be less than the angular extent of a single pixel. In this regime 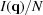 is independent of crystal size, within measurable limits, and the prefactor to the unit-cell transform can be safely approximated by
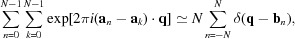
where 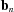 is the reciprocal-lattice vector with index *n*. Thus both terms scale linearly with *N* and are indeed quite comparable in terms of the number of scattered contributing photons (Chapman *et al.*, 2017 ▸).

**Figure 1:**
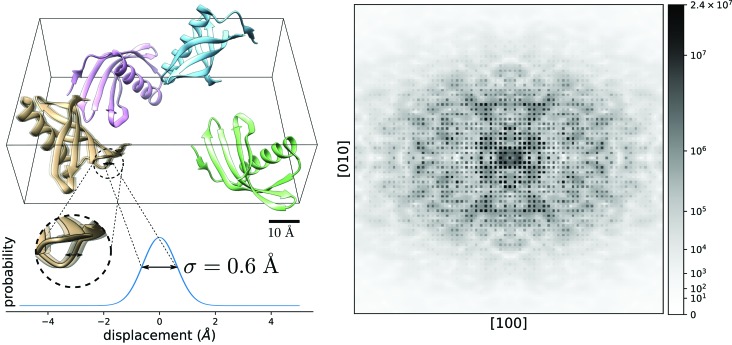
Model of a 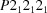 crystal exhibiting translational disorder of the rigid-unit locations and its diffraction. Left: ribbon diagram of a unit cell containing four rigid units (the potato multicystatin monomers), where we show the rigid-body translations for one of the rigid units to the left and right as a transparent underlay, corresponding to one standard deviation (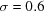
 Å). Right: central section through the diffraction volume of the crystal in the plane [*hk*0].

In Fig. 1 ▸ we show the simulated diffraction from a potato multicystatin crystal with translational disorder [PDB (Protein Data Bank) model 2w9q, Nissen *et al.*, 2009 ▸]. The space group is 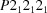, which is the most common for protein crystals (RCSB, 2018 ▸) [it occurs in roughly one-third of all monomeric proteins (Wukovitz & Yeates, 1995 ▸)]. Each unit cell in the crystal has four symmetry-related copies of the rigid unit. The crystal is simulated with a disorder length of σ = 0.6 Å and a crystal size 100 unit cells wide, with a volume of approximately 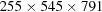
 nm. We should note that in fact such crystals are unlikely to be cubic in shape. In this case the Bragg reflections are around three orders of magnitude more intense than the continuous diffraction intensity per pixel for small scattering angles corresponding to the first few Bragg reflections (the colour scale in Fig. 1 ▸ has been truncated to show the continuous diffraction). At larger scattering angles the situation is reversed, such that the Bragg reflection intensities are negligible when compared with that of the continuous diffraction. The diffraction data are shown as a slice through the diffraction volume, intersecting the origin 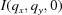, and the pixel sampling is chosen so that the Bragg reflections are centred on every second pixel along each dimension. This data set thus contains eight times the number of data points that would normally be stored in a list of Bragg peak intensities at the same resolution.

In this example we consider the simplest case, in which the rotation and translation operators that relate each of the rigid units to each other {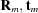} form the space group of the crystal. That is, the rigid units are related by the global crystallographic symmetry and not just by local (or pseudo-) symmetries. With respect to the information content of the Bragg reflections, this represents a worst-case scenario, in which the Bragg reflections and the continuous diffraction follow the point-group symmetry of the crystal with inversion symmetry (by Friedel’s law), in this case yielding the space group *Pmmm*. Consequently there are eight equivalent intensity values for most reciprocal vectors, excluding special values of 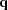 such as the origin. Any additional local pseudo-symmetries will only increase the information content, although the corresponding symmetry operations {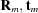} would need to be known (or determined) in order to benefit from this additional information. For the rest of this article, we will present the general form of this algorithm, applicable when the rigid units are related by crystallographic or pseudo-symmetries. However, in the former case it is possible to make use of the crystal symmetry to more efficiently calculate each update in the iterative algorithm.

In Appendix *A* [App appa] we describe the noise model used to simulate the diffraction intensities. There we also describe how σ may be determined directly from the crystal diffraction prior to phasing and how the number of rigid units in the crystal can be evaluated by examination of the distribution of continuous diffraction intensities and its deviation from ideal Wilson statistics. It is necessary to have good estimates of these parameters in order to relate the Bragg and continuous diffraction intensities as needed to recover the structure from the diffraction, as described in the following section. However, it is likely that the algorithm could be modified to iteratively refine initial estimates for these values.

## 3. Iterative projection algorithm   

Having described the observable quantities, namely the diffraction intensities 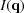, in terms of the quantity of interest which is the rigid-unit density 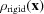, we now turn to the task of recovering 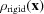 from 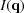 for a crystal with translational disorder. We assume that all quantities in equation (2) (except of course for 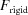) have been determined. This includes the disorder parameter σ, the internal symmetry of the unit cell (the 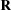
’s and 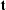
’s) and additionally the unit-cell parameters as well as the solvent fraction of the crystal. We cast this problem in the form of a phase problem in coherent diffractive imaging (CDI), which requires that we formulate projection operators responsible for enforcing the known constraints on the solution which are described below in Sections 4 and 5. We also describe the conditions that must be satisfied for a unique solution to exist in Section 6 and, finally, we verify that the rigid-unit density can be reconstructed from the simulated noisy diffraction intensity in Section 7.

The phase problem in CDI is commonly formulated as a set intersection problem in Euclidean space. For example, consider the problem of retrieving the structure of a single finite object from its diffraction intensities *I*. We can represent any 3D image as a point ψ in a vector space with a dimensionality equal to the number of voxels in the image. The value of each coordinate of ψ is given by the density of the object at the corresponding voxel. We can then define the set of all objects that are consistent with the given diffraction intensities (the data constraint set 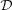) and the set of objects that are contained within a given finite volume (the real-space or support constraint set 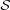). The solutions are given by the points ψ that form the intersection of the two constraint sets 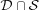, since these represent 3D images that are simultaneously consistent with the measured diffraction and the support constraint. The possible solutions can be related by trivial operations (Bruck & Sodin, 1979 ▸; Hayes *et al.*, 1980 ▸; Bates, 1982 ▸) such as inversion and translation. The projection operator 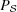 maps a given point ψ onto a point, in the set 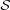, that is nearest to ψ, and similarly for 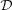. For example, 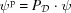 (the ‘

’ here simply connects the operator 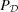 to the operand ψ) makes the smallest change to ψ necessary for 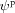 to be a member of the set 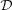 consistent with the measured data. Many algorithms, such as the error-reduction (ER), hybrid input–output (HIO) or difference-map (DM), repeatedly apply both of these projection operators to find the intersection points (Fienup, 1978 ▸; Bauschke *et al.*, 2002 ▸; Elser, 2003 ▸). For this work, we employ a combination of the ER and DM algorithms. The ER algorithm is simplest; it applies first the real-space projection followed by the data projection operator and is guaranteed to reduce the distance between the current guess (ψ) and the two constraint sets (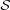 and 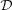) after each update but is prone to slow convergence or stagnation at points far from the global solution. The DM algorithm employs a somewhat more complex update rule that is designed to avoid stagnation and improve convergence speed but is more computationally expensive per update cycle, due to the increased number of projection operations required per iteration.

Before continuing, for notational convenience, let us first vectorize functions of 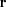 and 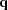 so that, say, 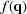 can be represented as a vector 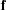 such that 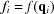 and all equations relating bold quantities should be understood as element-wise relations. For example 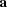 = 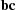 is equivalent to 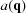 = 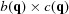 for all 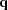. Note that we have used simple juxta­position, rather than ‘
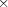
’, to represent element-wise multiplication in order to avoid confusion with the vector cross-product operator. In the above example, the support projection 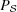 is carried out by setting all elements of 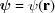 outside the support region 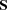 to zero, so that 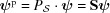, where 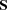 (not to be confused with the set 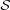) is 1 inside the support region and 0 outside.

## 4. Data projection   

For the data projection 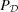, we now employ a useful property of projection operators, which is that they may be defined in real or reciprocal space. This is because the Euclidean distances between vectors are preserved under a Fourier transform (Parseval’s theorem). In this example, where diffraction is measured from a single finite object, the diffraction intensities of our object 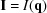 are equal to the square modulus of the Fourier transform of the object density which in turn is equal to the sum of the squares of its real and imaginary components 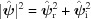, where 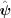 is the Fourier transform of 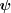. At every voxel in reciprocal space we wish to make the smallest change to the independent variables 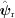 and 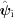 such that 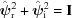. This equation describes the constraint surface at each *q* value as a circle of radius 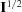 and the projection operator simply scales 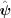 while keeping the ratio 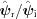 fixed: 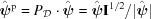.

Let us return to the case of a crystal with translational disorder. In this case it is not immediately clear how these projection operators should be defined. To see this, let us group the prefactors to 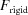 in equation (2) into the diffuse weighting terms 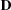 and the Bragg weighting terms 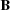:
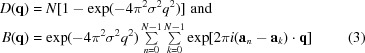
so that equation (2) can now be written:
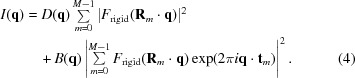
Consider the influence of 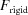 on *I* in our 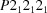 crystal. Here 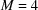 and so, by equation (4), each *q*-space voxel gains contributions from four different Fourier components of 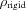 from each of the four orientations of 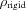. Conversely, each Fourier component of 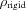 will influence the intensity observed in the four symmetry-related *q*-space voxels. Therefore, in order to determine the projection operation for a single Fourier component of 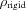, four coupled non-linear equations must be solved.

One way to decouple the effect of the symmetry-related values of 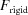 on the observed intensity is to expand the state vector to include each occurrence of 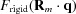 in equation (4) as an independent mode:
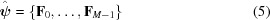
where
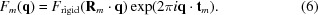
Inserting equation (6) into equation (4) yields
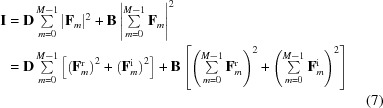
where, in the second line, we have expanded 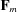 in terms of its real and imaginary components (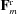 and 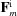, respectively) in order to better illustrate the number of independent variables.

Consider first when there is no crystal disorder and measurements are only taken at the Bragg peaks. Millane & Lo (2013 ▸) have examined this case, where 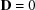. They set the coherent sum over the reciprocal rigid units equal to the unit-cell transform 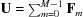. The constraint surface is now a circle in 2D space (for every voxel in reciprocal space) and the data projection must rescale 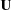 by the ratio 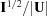. With this construction, the real-space projection enforces any known internal symmetries of the unit cell. Indeed, these projections can be used to phase Bragg reflections using the principles of CDI for crystals of high solvent content (He & Su, 2015 ▸).

At the other extreme, when 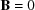, and the set of *M* rotation operators 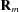 form a group, Elser & Millane (2008 ▸) have shown that the constraint surface forms a 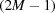-sphere in 2*M*-dimensional space of radius 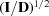, one dimension for each of the real and imaginary components of 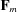 and again for each voxel in reciprocal space. The data projection then rescales each of the rotated 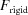
’s by the ratio 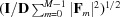. As a physical realization of this case, Elser and Millane were motivated by diffraction of laser-aligned molecules which can exist in equal populations aligned parallel and antiparallel to an alignment axis with completely random intermolecule translations (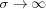).

In general, however, 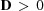 and 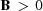, which is a departure from the above cases in two ways. First, the intensity depends on a mixture of coherent and incoherent additions over the 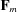. Second, the contribution to the intensity from the coherent and incoherent summations of 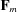 has weighting factors that can vary with 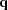. Chen *et al.* (2016 ▸) formulated projection operators to account for mixtures of coherent and incoherent additions, arising in the context of diffraction of finite crystals. However, this formulation can only incorporate constant (non-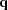-dependent) values for 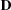 and 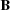. In the second case, we have a more fundamental departure from previous work in this field, where most phase problems rely on data projection operators that project a point onto a hyper-sphere or a hyper-cylinder. In the present case, however, equation (7) describes a 2*M*-dimensional hyper-ellipsoid for arbitrary 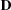 and 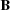. For a crystal composed of a single rigid-unit type, this *2M*-hyper-ellipsoid can be reduced to a 2D ellipse (in general, the dimension of the ellipse is twice the number of rigid-unit types). Nevertheless, the projection cannot be described in terms of simple operations (such as rescaling). In the following section (4.1) we derive the data projection operator 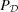 and show that it satisfies the requirements as a distance-minimizing mapping of 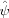 onto the set 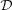. This involves the use of a simple algorithm for projecting a point onto an ellipse surface, for which we have written a Python implementation of the procedure described by Eberly (2011 ▸).

### 4.1. Data projection: derivation   

Given the state vector 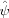, the data projection 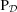 is an operator that minimizes the Euclidean distance: 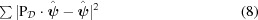
such that equation (7) is satisfied by 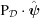 (the sum is over each element of 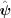).

Currently, equation (7) describes a multi-dimensional ellipse; this we know simply because it is a quadratic equation constraining each of the values in our state vector 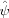 (or equivalently the set of 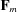
’s). One way to simplify equation (7) is to rotate our basis vectors so that they are aligned to the principal axes of the ellipse. Fortunately, this rotation matrix is somewhat trivial to construct in the present case. Consider the second term in equation (7), which involves the coherent sum over all *m* components of 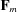, suggesting that we might find a rotation matrix such that this coherent sum is represented by a single component in the new basis. Indeed, 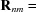 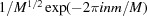 is sufficient for this task and 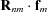 is nothing but a discrete Fourier transform with an easily constructed inverse 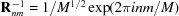.

So, let us rotate our state vector from 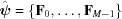 to 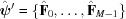, where 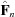 is the Fourier transform of 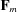 over *m* (not 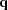
!), so that
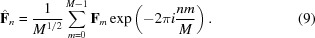
With this transformation 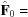 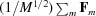 and 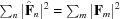. Equation (7) becomes 
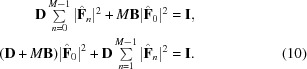
Note that 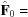 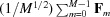 is nothing but the coherent sum over each rigid unit in the unit cell (the unit-cell transform of the crystal) scaled by 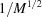. The transform from 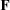 to 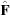 is unitary on our state vector and so distances between vectors in this space are preserved. As a consequence 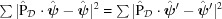. That is, we are free to rotate our state vector from 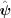 to 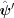, apply the data projection to obtain 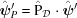 and then rotate back to get the projected state vector 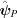 in our original basis.

With the substitutions 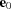 = 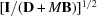 and 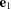 = 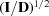 we can recast equation (10) in the form of a 2*M*-dimensional hyper-ellipsoid (one dimension for each of the real and imaginary components of 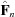):
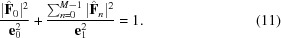
Here all of the hyper-ellipsoid semi-axes (at a particular voxel) are one of 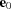 or 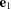, suggesting a high degree of symmetry, which we will now make use of. First we note that the phases of 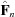 are not present in the equation for the hyper-ellipsoid (we remind the reader that these are not the phases of the scattering amplitudes which we are trying to solve for). Therefore, a change in these phases represents a motion in 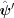 that is parallel to the surface of the hyper-ellipsoid. Since the vector 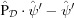 that projects 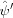 onto the constraint surface must be orthogonal to the constraint surface, this vector must also be independent of these phases. We can therefore keep the phases of 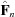 constant in our projection and factor them out of equation (11). The same is also true for any relative change in 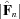 (for 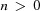) that keeps the total (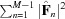) constant. Therefore we can make the substitution:
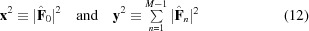
so that equation (11) reduces to the equation for a 2D ellipse:
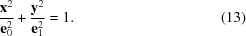
Thus the data projection for the state vector 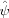 maps to the problem of projecting any 2D vector (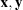) to the closest point on the surface of the ellipse 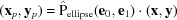.

Although we can find no closed-form solution for this projection, the points (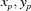) can be obtained by assessing candidates from the roots of a fourth-order polynomial equation (Hart, 1994 ▸). In an excellent review by Eberly (2011 ▸), this method is compared to numerical solutions based on root finding. He finds that the bisection method applied to a parametrized form of the ellipse equation provides the most reliable results, and can be generalized to any number of dimensions. We provide Python code that projects a point onto an ellipse surface following the suggestions of Eberly.^2^

While this may be the first time that a physical diffraction model has motivated the use of an ellipse projection, Borwein *et al.* (2018 ▸) have developed an algorithm for projecting a point onto a 2D ellipse for the purpose of analysing the dynamics of an iterative algorithm called the Douglas–Rachford method. They employ an algorithm based on Newton’s method (a root-finding algorithm), an approach that Eberly had earlier rejected in favour of the bisection search (because it is more numerically stable). Shortly before this work, Elser (2017 ▸), also in the context of phase retrieval, developed algorithms for projecting a point onto constraint surfaces that can be described by the matrix equation 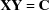, where 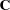 is the constraint matrix and 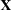, 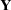 contain the state variables. While this constraint equation cannot be used to describe an ellipse, the iterative scheme employed by Elser to solve for these other projections is applicable in the present case. We have tested the algorithms from both Elser and Eberly (though we do not claim to have done so definitively) and found that they are roughly equivalent in speed and robustness. However, we favour the approach described by Eberly because it is well documented.

**Figure 2:**
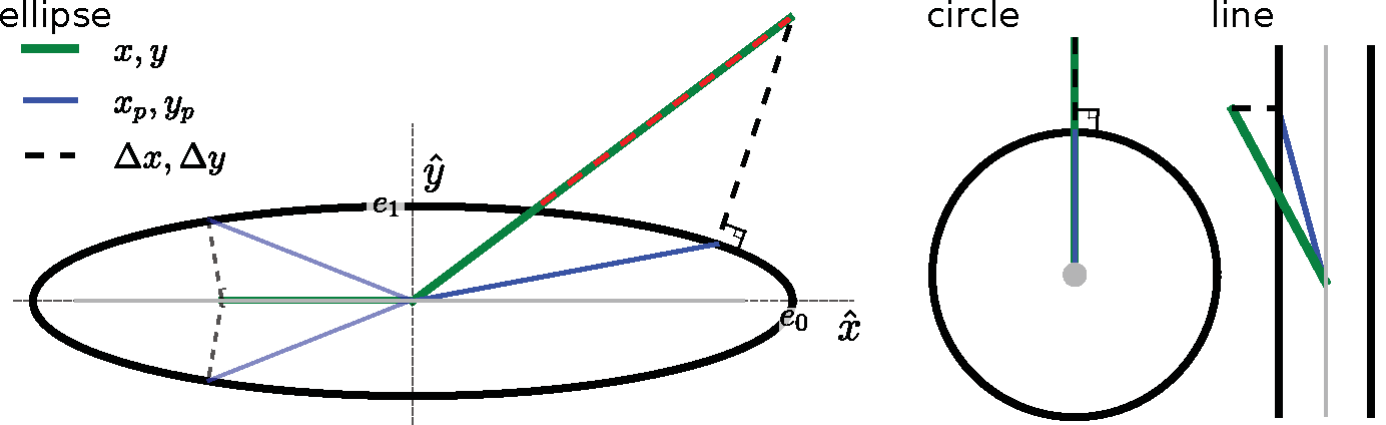
*Elliptical* data projection of the diffuse and unit-cell amplitudes onto the data constraint surface with 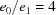; the red dashed line illustrates the projected path taken by a simple rescaling and the black dashed line the shortest path to the constraint surface. *Spherical* projection onto a circle with 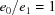. *Line* projection onto a line along the axis with 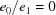.

As an example, consider an ellipse with 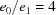, as shown to the left in Fig. 2 ▸. The black line is the set of all points (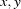) that are consistent with the measured intensity at a given 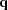. Starting at a given point (shown in green), the data projection finds the closest point on the ellipse (shown as the blue vector) where 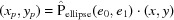. For an initial point 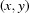 along the major axis of the ellipse in the interval 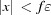 (shown in grey), where *f* and ∊ are the focus [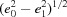] and eccentricity (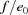) of the ellipse, respectively, the projection operation has two possible outcomes 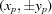 (as shown in lighter blue). In this case our algorithm arbitrarily chooses to project upwards to 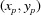. If 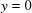 and 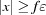 then *x* is projected to the right- or left-most point of the ellipse, *i.e.* 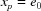 for 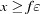 and 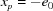 for 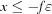.

In contrast to this data projection, consider conventional phase retrieval with a single coherent mode ψ; here the data projection is given by 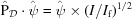 where 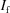 is the forward model for the measured intensity, given the current state vector (usually 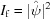). This is a simple rescaling of the state vector by the ratio of the square root of the intensity with the forward model of the intensity and is illustrated by the red dashed lines in Fig. 2 ▸. This is not the closest point on the constraint set to 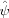, and hence is not a projection operator, and therefore an iterative algorithm based upon this will not possess the standard convergence properties.

In Fig. 2 ▸ (middle) we show the special case where 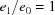 and the ellipse reduces to a circle. In the unlikely case where this applies, the data projection reduces to a rescaling of the model intensity and the elliptical projection is identical to the conventional projection. When 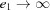 (right) or 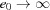 (not shown) the data projection rescales *x* (right) or *y* (not shown) with two solutions along the axis.

Having projected 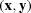 onto the ellipse, the data projection then simply maps the points 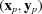 back into our original basis. This is achieved by rescaling 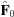 by the ratio 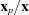 and each of the 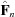 by 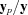, for 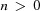, and then computing the discrete inverse Fourier transform over *n*.

In Table 1 ▸ we summarize the procedure for performing the data projection on each of the Fourier space modes 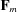.

## 5. Real-space projection and support update   


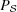
 is more straightforward to construct; it makes the smallest change to a given estimate for the rigid-unit densities at a given iterate such that the mapped projection is consistent with our prior knowledge of the crystal. We must ensure that the rigid units are all identical copies of themselves (in different orientations), that they are arranged according to the symmetry of the crystal, that their densities do not overlap, and that they each have a given number of volume elements that deviate from the solvent density level, consistent with the solvent fraction of the crystal.

In the following section (5.1) we derive 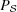 and show that 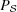 also satisfies the requirements as a projection operator. To summarize: the *M* estimates for the rigid units are averaged within the volume known to occupy the rigid unit, that is the ‘support volume’, after first overlaying them by applying the inverse of the rotation and translation operations for each. This averaged rigid unit is then replicated and placed back into the unit cell according to the symmetry of the crystal. These *M* copies of the rigid unit are then propagated back to reciprocal space by a Fourier transform.

Additionally, if the support volume is not known, then it can be periodically updated based on the current estimate of the rigid-unit density in a manner similar to that of Marchesini’s ‘shrink-wrap’ algorithm (Marchesini *et al.*, 2003 ▸). In the current case of the potato multicystatin crystal, an estimate for the support was updated by keeping the highest density values for the averaged rigid unit, within a loose support region, such that the total number of elements is equal to a given number (the voxel number support) consistent with the solvent fraction of the crystal. This support volume is then convolved with a Gaussian kernel and the voxel number support is applied once again to this function. For this first step we have found that it was necessary to apply the additional (very loose) support on the rigid-unit density. This region is indicated by the black dashed line in Fig. 5 (bottom left) and is equal to 40% of the unit-cell volume. Without this additional constraint it was commonly observed that the support would become fragmented, even with an aggressive smoothing parameter. The Gaussian smoothing kernel has a standard deviation of 0.5 Å.

### 5.1. Real-space projection and support update: derivation   

Let us now formulate the constraints listed in the previous section mathematically: we wish to find 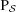 such that
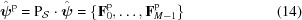
where
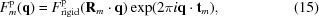
ensuring that the rigid-unit densities are identical and arranged according to the crystal symmetry. We also require that
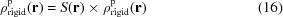
where 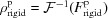 and 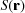 is the support of the rigid unit with a given volume, ensuring that the rigid units have a fixed number of voxels. Note that equation (16) defines a constraint that must be enforced by the projection operation and is not (as is often the case) the projection operation itself, which we will derive shortly. So, to satisfy equation (16) we require that
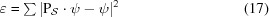
is minimized for any ψ, ensuring that 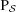 is a distance-minimizing projection in Euclidean space. Note that in equations (14)–(16) we have used the superscript ‘p’ to represent projected quantities. In addition we demand that there is no spatial overlap between the symmetry-related copies of the rigid unit in the crystal. To reiterate, the goal here is to find 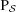, which is defined by the projected modes 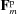, which in turn are given by 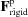 (related by a Fourier transform to 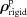). This can be achieved by minimizing equation (17) with respect to 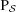.

First, let us assume that the support region 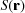 is known. We can express the error in equation (17) in terms of the deviation between the real-space rigid units inside this support region. Expanding equation (17) in terms of the unit-cell modes yields
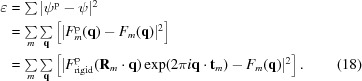
As the distance between vectors is preserved under a unitary transformation of the vectors, we are free to apply the following transformations:
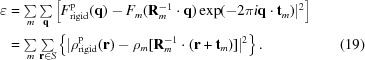
In this first step we have applied the inverse of the rotation and translation operators defined by the space group of the crystal to each of the unit-cell modes. This serves to bring each estimate of the reciprocal rigid unit into register. In the following step we propagate each mode to real space via an inverse Fourier transform where the sum over 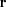 is confined to the real-space volume of the rigid unit such that 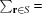 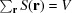.

It can be shown that
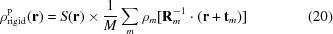
minimizes the Euclidean distance (∊) in equation (19) (Bricogne, 1974 ▸). 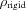 in equation (20) now satisfies two constraints, the internal symmetry of the unit cell and the support constraint, and is thus at an intersection of these two sets. Because the two projections, multiplication by 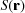 and the average over *m*, commute they form a single projection operation onto the set formed by their intersection.

**Figure 3:**
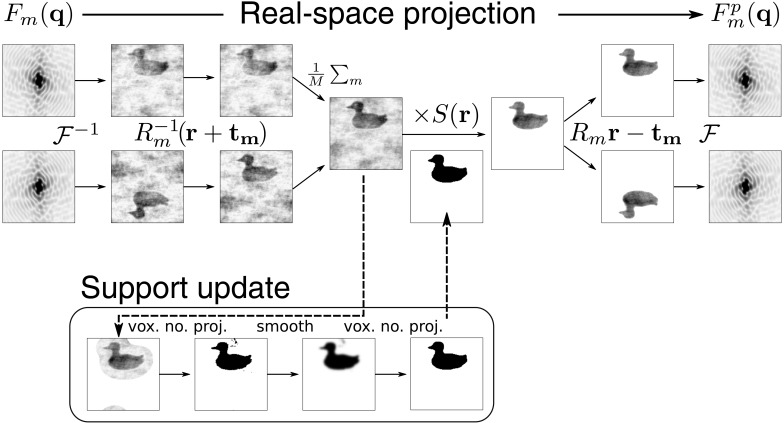
Flow diagram illustrating the real-space projection operation for a 2D crystal. The crystal has the space group 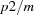 and the unit cell consists of two ducks separated by a mirror plane cut horizontally across the middle of the array. Also illustrated is the procedure for updating the real-space support region, which is not part of the projection operation.

**Figure 4:**
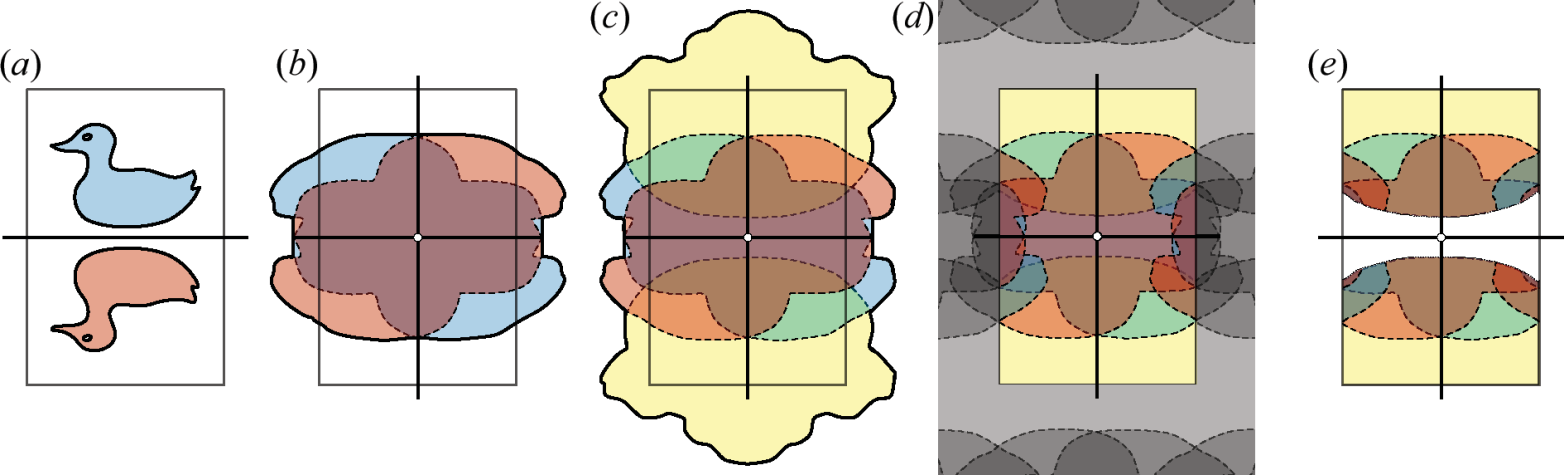
(*a*) A unit cell with two identical rigid units (ducks) related by a mirror line (horizontal line); the border indicates the unit-cell dimensions in the *pm* crystal. (*b*) The symmetry summed autocorrelation region of the two ducks shown in (*a*) (solid line) with space group 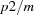, with regions corresponding to the two autocorrelation functions coloured to match the corresponding duck. Inversion symmetry through the origin (white circle) has generated a second mirror line (vertical line). (*c*) The full un-aliased autocorrelation of the unit cell, with the cross-correlation terms between the two ducks coloured in yellow. (*d*) The Patterson map of the crystal inside the unit-cell area (coloured) and outside the unit cell (in grey). (*e*) The Patterson map of the crystal confined to the unit-cell area and excluding the region occupied only by the aliased autocorrelation of the two ducks.

We can now simply Fourier transform 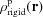 to obtain 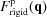. The projected modes are then given by application of equation (15). These operations are illustrated as a flow diagram in Fig. 3 ▸, where we have used a 2D crystal of ducks with the space group 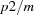. This is the same toy model as illustrated in Fig. 4 ▸.

Now we describe our procedure for updating the support region 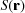, given an estimate for the rigid-unit density 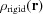. This procedure consists of four steps. First, 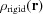 is multiplied by a very loose support 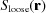. This region may be much bigger than the rigid unit itself and may also contain parts of the unit cell which are occupied by the symmetry-related copies of the rigid unit. We found that this step is necessary to avoid fragmentation of the support to different regions of the field of view, despite the aforementioned smoothing procedure. Second, within the loose support region, we apply a voxel number projection which enforces the solvent fraction of the crystal. Third, this support volume is then smoothed with a Gaussian kernal. This step, which is employed in a similar way in Marchesini’s ‘shrink-wrap’ algorithm (Marchesini *et al.*, 2003 ▸), biases low-resolution features in 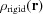 and helps to remove small isolated regions from the resulting support envelope. Finally, the voxel number support is applied (once again) to the smoothed support volume.

In both cases the voxel number support, first posited (in the context of CDI) by Elser (2003 ▸), has been modified to include collision avoidance between rigid units in the crystal:

(1) For all 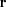, assign 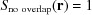


if 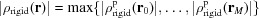


and 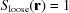,

otherwise assign 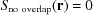.

(2) Within the no overlap volume [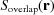] keep only the *V* most intense values of 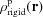 by setting:


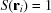
 for 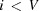


where the set 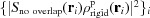 is sorted from highest to lowest value,

where 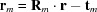.

This last operation is, of itself, a projection operator (Elser, 2003 ▸) but does not commute with the averaging projection and so the procedure outlined here for updating 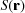 cannot join equation (20) as a single projection and should therefore be applied periodically outside the projection algorithm.

And so, with the above procedure for finding the support volume and equation (20) for the rigid unit, we can map the unit-cell modes onto the closest set of modes that are consistent with a single rigid unit.

## 6. Uniqueness of the solution   

In phase retrieval the constraint ratio (Ω) is defined by the ratio of linearly independent equations to unknown quantities in the phase problem^3^ (Elser & Millane, 2008 ▸). If 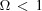 then the phase problem is certainly under-determined and there is no unique solution. For 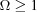, a given solution may be unique and in some cases it can be shown that multiple solutions are pathologically rare (Bates, 1984 ▸). Thus 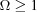 is a necessary but not sufficient condition for a unique solution. A single isolated object has 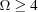, where the lower bound corresponds to an object with a convex and centrosymmetric support, while non-convex supports have a higher constraint ratio and are easier to solve (Fienup, 1987 ▸).

In the following section (6.1) we derive expressions for the constraint ratio when phasing from Bragg reflections (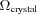 corresponding to 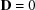), continuous diffraction (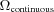 for 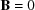) and from their sum (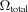 when 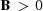 and 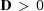). We find that for the ten most common crystal space groups released in the PDB, representing approximately 77% of all structures in the PDB (RCSB, 2018 ▸), 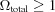, suggesting that *ab initio* phasing is almost always possible in principle for crystals that possess purely translational disorder.

These results are summarized in Table 2 ▸ where we provide the lower bound of these constraint ratios for a few crystal space groups, including the ten most common space groups listed in the PDB (RCSB, 2018 ▸). These lower bounds correspond to the case of zero solvent fraction. Most proteins have a significant volume of solvent which, if known or determined, will increase Ω.

### 6.1. Uniqueness of the solution: derivation   

The Fourier transform of diffraction intensities of any object (including a crystal or single particle) is equal to the autocorrelation function of that object. Thus, the information content of a diffraction pattern can be quantified by the area and symmetry of the non-zero regions of the autocorrelation function. In phase retrieval the constraint ratio (Ω) defines the ratio of independent equations to unknown quantities in the phase problem. For a single isolated object 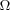 = 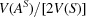, where 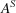 is the support of the autocorrelation of the object support, that is, the region outside of which *A* is known to be zero, 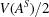 is half the volume of the 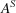 and 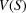 is the number of unknown elements (voxels) in the object support (*S*) (Elser & Millane, 2008 ▸). The factor of one-half arises because the autocorrelation of the object is equal to the inverse Fourier transform of the real-valued diffraction intensities and thus has Hermitian symmetry, 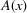 = 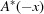 = 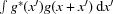 = 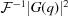 where 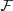 is the Fourier transform operator and 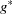 is the complex conjugate of *g*. If the object function is complex valued, then the number of unknowns is twice the support volume 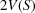, but in that case *A* is also complex, yielding twice the number of equations and so Ω is unchanged. A convex and centrosymmetric object (such as a cuboid) has 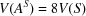 and yields the lowest constraint ratio with 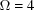, while non-convex supports have a higher constraint ratio and are easier to solve (Fienup, 1987 ▸).

Since the continuous diffraction is the incoherent sum of the transforms of the rigid units in each of their orientations of the crystal [see equation (2)], the inverse Fourier transform of the continuous diffraction is the sum of the autocorrelations of each of these rigid units. By way of illustration, consider a single unit cell in a 2D crystal with plane group *pm* as shown in Fig. 4 ▸(*a*). The unit cell consists of two rigid units (here ducks) which randomly displace independently of each other. The dimensions of the unit cell are indicated by the black rectangle and the single mirror plane is indicated by the horizontal thick line. The regions occupied by the two symmetry-related ducks we write as 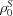 and 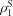 where 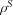 is the support area of ρ and the subscript is used to index the rigid unit in the unit cell (0 for blue and 1 for red). In Fig. 4 ▸(*b*) we display two regions, each corresponding to the support area of the autocorrelation of one of the ducks (the colouring indicates which is which). The rigid outline bounds the union of the two regions which is given by 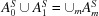. Here inversion symmetry at the origin (shown as a white circle) has generated a second mirror plane perpendicular to the first and so the unique area of 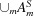 is confined to one-fourth of the total (rather than one-half as above). In this case the constraint ratio is therefore less than that given by diffraction from a single object by a factor 2, such that 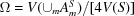. For the general case, the constraint ratio from symmetry-averaged diffraction data has been examined in the work of Elser & Millane (2008 ▸). They find that when the set of *M* orientations (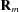) form a closed set (they form a group), then the constraint ratio is given by
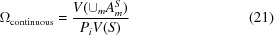
where 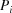 is the number of symmetry operators in the space group (including the identity operator) generated by inversion through the origin and the set of rotation operators 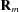 (this is equal to the number of symmetry operators in the Patterson group). Thus 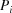 is always greater than or equal to 2. In the worst case, the support of the object is centrosymmetric and invariant to a rotation under any of the rotation operations, in which case the autocorrelation functions all overlap and 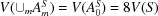, so that 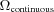 = 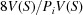 = 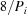. That is, the constraint ratio is reduced by a factor equal to the number of point-group operations (excluding inversion symmetry) with respect to the single-particle case. For the simulation shown in Fig. 1 ▸ the space group is 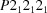, this has a Patterson group *Pmmm* which has eight symmetry operations including inversion through the origin, yielding 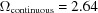. If the support were (say) a sphere, then 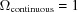, in which case phase retrieval is generally not considered to be possible in the absence of other prior constraints [that is, beyond a knowledge of *S* or 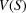].

As seen in equation (2), the Bragg peak intensities are given by the modulus square of the Fourier transform of the unit cell. That is, it is the coherent addition of all rigid units, arranged and oriented in the unit cell. Thus, the autocorrelation of the unit cell contains autocorrelations of the two rigid units (as is the case for the continuous diffraction) in addition to cross-correlation terms that arise from the quadratic expansion of the autocorrelation in terms of the two rigid units: 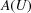 = 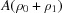 = 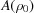 + 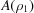 + 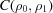 + 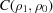, where 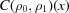 = 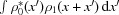. The autocorrelation support of the unit cell (bold outline) including the cross-correlation supports (yellow region) and the two autocorrelation support regions [red and blue as in (*b*)] are shown in Fig. 4 ▸(*c*). This function has the same symmetry axes as those in (*b*) and has a larger support that also extends beyond the region of the unit cell itself. The inverse Fourier transform of Bragg peaks from a perfect crystal is equal to the autocorrelation of the entire (perfect) crystal, which has the same periodicity in real space as the crystal. Therefore, the autocorrelation of the single unit cell shown in Fig. 4 ▸(*c*) overlaps with the neighbouring cells, giving rise to an aliasing. This aliased autocorrelation function is called the Patterson function of the crystal. This aliasing is illustrated in Fig. 4 ▸(*d*). To guide the eye, regions that are related to those within the unit-cell area by translation symmetry are shown in grey. We write the autocorrelation function, aliased by the reciprocal lattice [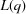] and bounded by the unit-cell support [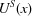], as 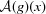 = 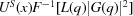, the aliased autocorrelation support for the *m*th rigid unit as 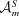 and the aliased cross-correlation support for rigid units *m* and *n* as 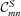. As the Patterson map possesses the same symmetry as the autocorrelations in Fig. 4 ▸(*b*) and the number of unknowns are also the same, the expression for the constraint ratio is given by equation (21) but with the substitution 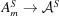: 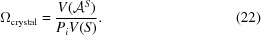
This derivation follows closely that of Millane & Arnal (2015 ▸). There they also consider the case when only the solvent content [and consequently 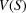] is known rather than the support itself. They find that when the volume, and not the envelope, of the rigid unit is used to constrain the phase problem, then the constraint ratio remains unchanged, although the speed of convergence is much reduced due to the large multiplicity of supports with equal volume. Consider the extreme case where the space group of the crystal and the rigid object support are unknown; then the number of unknowns is equal to the volume of the unit cell 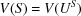, 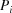 is given by the identity operator and the point-group symmetry of the Patterson map so that 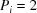, 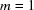 and 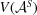 is also equal to 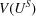. This gives 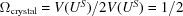 [as discovered by Sayre (1952 ▸)] and is a factor of eight less than the worst case for single-molecule imaging. For a 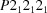 crystal and with no support volume, 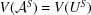, 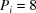 and 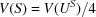 (since the four rigid units must fit within the unit cell) once again give 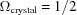. In both cases 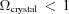 and so phase retrieval from Bragg reflections alone and without knowledge of the solvent content is not feasible without other constraints. In general, the number of symmetry operations in the Patterson symmetry is equal to one or two times the number of symmetry operations in the crystal, so 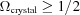 for crystals without inversion symmetry in the crystal point group and 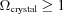 for crystals that already possess inversion symmetry in the corresponding point group (*i.e.* the Patterson map possesses the same number of symmetry operators as the crystal itself).

The constraint ratio will increase when a tight support for the rigid unit is known, which is possible when the solvent content of the crystal is not negligible, or when some of the 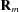 are not members of a closed group (*i.e.* there are rigid units related by local pseudo-symmetry), by increasing 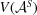. This is illustrated in part by the constraint ratio for the simulation shown in Fig. 1 ▸ (again with a tight support) where 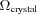 increases from 1 to 1.38 due to the solvent fraction.

Finally, we now consider the case where the diffraction is given by the weighted addition of the Bragg reflections and the continuous diffraction. For large crystals illuminated by coherent radiation the Bragg peaks are effectively point like, while the continuous diffraction produces smooth diffraction features (sometimes called speckles) which are band limited due to the finite extent of the autocorrelation function. Thus, if the crystal diffraction is sufficiently sampled then the continuous diffraction for points on the reciprocal lattice can be determined by Fourier interpolation of the neighbouring values. The continuous diffraction and the Bragg reflections are then separable and can be demodulated by the known weighting factors, although in practice measurement error will prevent perfect separation. One can also think of this process in autocorrelation space: the inverse Fourier transform of the diffraction will yield the autocorrelation of the rigid units located in the centre of the array plus the Patterson map which repeats on the crystal lattice. Because of the oversampling at least two periods of the Patterson map will be contained within the bounds of the array in each direction and so the central region of the Patterson map can be determined by neighbouring cells and thus subtracted from the global function to give the autocorrelation due to the continuous diffraction alone.

One might think that in such a case the constraint ratio is then given by the sum 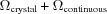 since they share a common denominator. However, not all points within the Patterson map are linearly independent from those in the symmetry summed autocorrelation. One can see in Fig. 4 ▸(*d*) that there is a region near the origin of the Patterson map wherein the aliased autocorrelation of the rigid units does not overlap the set of cross-correlation terms, given by the unwieldy expression 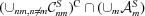 (the superscript ‘C’ denotes the complement of a set). All points that lie in this region of the Patterson map can be generated by the symmetry summed autocorrelation functions. This can be achieved by subsampling the symmetry summed autocorrelation *A* [as shown in Fig. 4 ▸(*b*)] in Fourier space on the reciprocal lattice to form 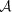 [the aliased symmetry summed autocorrelation shown near the centre in (*d*)]. Therefore, we must exclude this region from the Patterson map before adding the region occupied by the symmetry summed autocorrelations. This is easily achieved by confining the Patterson map to the regions where the cross-correlation terms are non-zero. This region is shown in Fig. 4 ▸(*e*) and is just the region occupied by the aliased cross-correlation terms 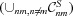:
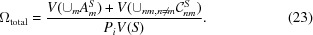
For a tightly packed crystal, the rigid units will be in close contact and thus the aliased cross-correlation regions will fully overlap the aliased autocorrelation regions in the Patterson map. In that case 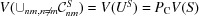, where 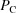 is the number of symmetry operations in the crystal space group. In this case no region of the Patterson map can be generated from the symmetry summed autocorrelation and so there is no redundancy in the information provided by the Bragg reflections and the continuous diffraction, leading to 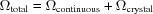. In the worst case, for a convex and centrosymmetric support, 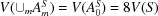. Thus the total constraint ratio always satisfies 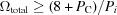. As we have mentioned previously 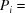 (1 or 2) 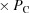 and so 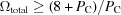 or 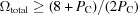, depending on the space group of the crystal.

## 7. Simulation results   

Now that we have defined the crystal diffraction model, determined the required projection operators and that a unique solution may exist, we now demonstrate that our IPA is capable of solving for the electron density of a potato multicystatin crystal from simulated noisy diffraction.

In the absence of noise, with a fixed tight support volume and with no error in the input σ value (or form of 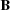 and 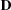) the electron density of the potato multicystatin monomer, whose model is shown in Fig. 1 ▸ (left), can be retrieved to within numerical precision. From a random start this typically occurs within the first 100 iterations of the DM algorithm. However, when the diffraction is noisy and the shape and position of the rigid unit are not given to the algorithm but instead only a loose support and the crystal solvent fraction are provided, then many more iterations are required for convergence (6000 in this case).

**Figure 5:**
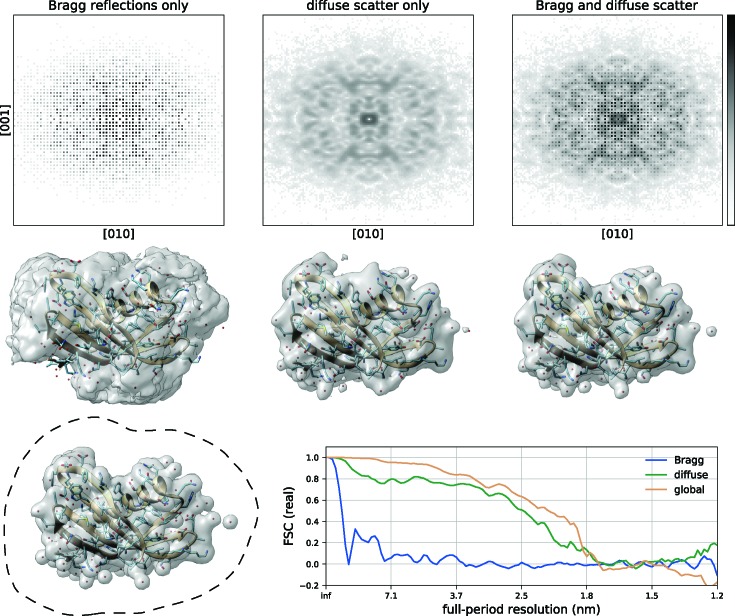
Rigid-unit reconstructions from the Bragg reflection intensities (left), the diffuse scatter (middle) and the full diffraction intensity including the sum of both the Bragg reflections as well as the diffuse scatter (right). Top row: noisy diffraction intensities used for the reconstructions, in the (100) plane shown with the same log-scale colour map. Middle row: the corresponding reconstructions of the rigid unit shown as one-level contour plots overlaid on the potato multicystatin monomer model (for visual reference). These images were made using the UCSF *Chimera* software package (Pettersen *et al.*, 2004 ▸). Bottom: one-level contour plot of the ground-truth density (left). The real part of the FSC of each of the three reconstructions with the ground truth, as a function of the full period resolution (right).

In Fig. 5 ▸ we compare the rigid unit reconstructed from three simulated data sets. The three data sets are derived from the full 3D merged diffraction data as shown in Fig. 1 ▸: the contribution from the Bragg reflections alone 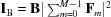 (left), the diffuse scatter alone 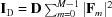 (middle column) and the full combined data set 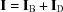 (right column) equal to the incoherent addition of the first two data sets. The total number of photons used to simulate the noisy diffraction intensities are 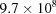, 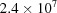 and their sum 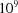, respectively. The number of photons was chosen such that the signal level drops to nearly zero at the highest diffraction angles covered by the detector. This was done to test the behaviour of the algorithm across a broad range of signal-to-noise levels. In each case the resulting rigid unit is the average of 50 independent reconstructions, starting with density values drawn from a uniform random number in the range 0 to 1 within the loose support volume (the outline of this volume is the black dashed line in the bottom left). The overall scale of the initial estimate is irrelevant here; after the first application of the data projection operator the scale is set by the diffraction intensities. But the random initialization helps to provide an unbiased solution and to avoid pathologies that might arise during the reconstruction from other simpler starting values (*e.g.* all zeros). The reconstruction algorithm is also the same in all three cases except that the weighting parameters for the Bragg and continuous diffraction have been set to zero where appropriate; see equation (3) for the definition of these weighting factors and Table 3 ▸ for more detailed parameters. These reconstructions are not molecular replacement solutions, and there is no model at all. That is, we are presenting (simulated) experimental phasing with no knowledge about chemistry.

The reconstructed rigid units corresponding to each of these diffraction intensities are shown in the middle row as single-level contour plots overlaid on top of the atomic model (again this model is not used for the reconstruction) for the potato multicystatin monomer, serving as a visual aid to the reconstruction quality. The contour level is set to an electron-density value of 0.2 e Å^−3^. The initial support was generated by thresholding the random initial guess until the specified number of volume elements for the rigid unit was obtained (the voxel number support projection). Subsequent updates to the support followed the recipe described in Section 5.1, maintaining the correct solvent fraction at every iteration.

The constraint ratio from the Bragg diffraction is 0.7 (as listed in Table 2 ▸) and so, being less than 1, we could not expect to reconstruct the rigid unit without the use of additional constraints. On the other hand, the constraint ratio of the continuous diffraction is 1.9, indicating that it should be possible to retrieve a unique solution and one can see this is borne out by the fidelity of the two reconstructions shown in Fig. 5 ▸. One can also see that although the reconstruction from the Bragg reflections alone has clearly failed, the reconstruction quality marginally improves when they are added to the continuous diffraction, consistent with the increase in the constraint ratio from 1.9 to 2.6. Although an increase in reconstruction quality is desirable, we suggest that the main benefit to the global reconstruction approach may be the fact that the Bragg and continuous diffraction need not be treated separately. Separating these components is otherwise a feat that could prove difficult due to the very large deviations in intensity between the Bragg and continuous diffraction at low scattering angles.

In order to quantitatively compare the reconstructions, we calculate the fidelity error which is a measure of the agreement with the ground truth, where 0 corresponds to perfect agreement and 1 to very poor agreement. In the present case the fidelity errors are 0.78, 0.44 and 0.26, respectively. Another measure of the reconstruction fidelity is the Fourier shell correlation (FSC) (Frank, 2006 ▸) which we plot in Fig. 5 ▸ (bottom right) for each of the three reconstructions. This shows a consistent increase in the FSC for the global reconstruction for most resolution shells (at high scattering angles the reconstruction is dominated by noise). Global reconstructions performed without noise added to the diffraction intensities are able to reach agreement with the ground truth to 1 part in 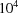. Both the fidelity error and the FSC are defined in Appendix *D* [App appd] [equations (26) and (27)].

## 8. Discussion and conclusion   

Having shown that model-free phasing of diffraction from crystals with translational disorder is possible, we now consider some aspects of the application of this method to experimental data. Because Bragg peaks often yield very bright and sharp peaks on the detector, any underlying background can usually be estimated (and thus subtracted from the data) by examining the detected signal in the immediate neighbourhood of the diffraction spot. This is not true however for the continuous diffraction. In general, this method places higher demands on data collection and estimation of the background, for example due to the crystal solvent, ice formation or from the carrying medium of the crystal such as a liquid jet, aerosol or sample holder. Chapman *et. al.* have recently suggested a method to estimate this background (Chapman *et al.*, 2017 ▸). Standard crystallographic methods for structure retrieval are also fairly robust with regard to missing diffraction intensity measurements. For instance, when calculating the *R*-free metric, some reflections are excluded when fitting the molecular model to the diffraction data (Brünger, 1992 ▸). However, in model-free phasing, missing data regions can lead to unconstrained modes in the reconstruction (Thibault *et al.*, 2006 ▸) which can be a problem, particularly near the origin where a beamstop is often placed. For these reasons, we expect that a combination of our proposed method with model fitting and refinement may often be the more robust approach, particularly for structures where prior information is available.

In this work we have assumed that the translational disorder is isotropic, with the displacements following a normal distribution. However, this is not required for the reconstruction algorithm and this procedure could likely be modified to account for alternative models for the rigid body’s translational motion. Indeed, all that is required is that the diffraction is partitioned into a coherent and incoherent sum over the rigid-unit transforms. The elliptical projection remains valid for arbitrary weighting functions.

However, for some crystals, translational disorder will not be the only significant contribution to the continuous diffraction. Other rigid-body motions of the rigid unit may be dominant or at least significant, and need to be accounted for to obtain an accurate description of the crystal diffraction. Extending the current work to account for these effects will greatly increase the number of potential structures that could be solved by our method and is a matter of ongoing research.

We hope that this work will soon lead to model-free phasing of crystals with unknown structures, particularly for those without a good reference. To that end, we have included code that, although not intended as a general application, should at least aid in the reproduction of our results and accelerate real-world applications for the work presented here.^4^

## Figures and Tables

**Figure 6:**
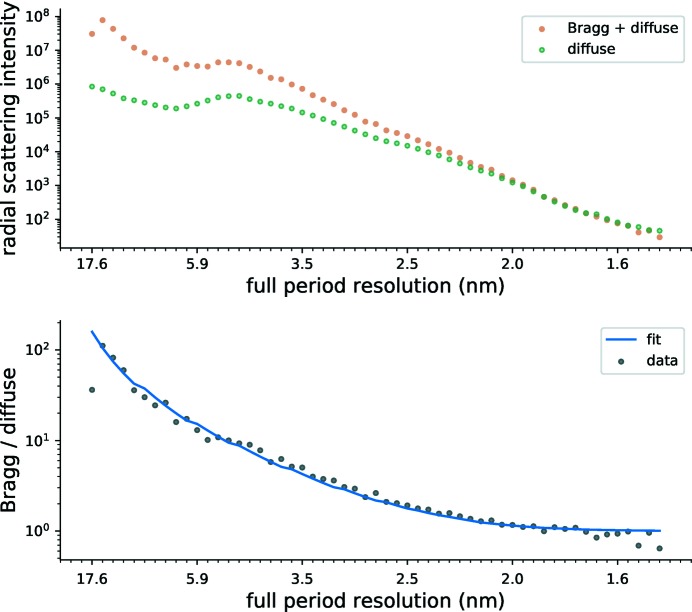
Top: radial profile of the scattering intensity on and off the reciprocal lattice, labelled ‘Bragg + diffuse’ and ‘diffuse’, respectively. Bottom: ratio of the on-Bragg to continuous diffraction shown on the top (black circles) and the model fit to this profile (blue line).

**Table 1:** Data projection operation The superscript ‘p’ signifies a projected quantity, 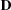 and 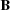 can be determined from the data and are defined in equation (3), and 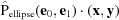 is calculated numerically.

Step 1	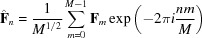
Step 2	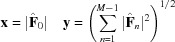
Step 3	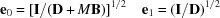
Step 4	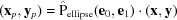
Step 5	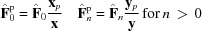
Step 6	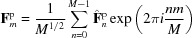

**Table 2:** The constraint ratio for *ab initio* phase retrieval from symmetry summed diffraction (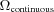), Bragg reflections (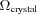) and from their sum (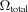) The lower limit corresponds to cases where there is no solvent content, the rigid-unit support is centrosymmetric and convex, the Bragg reflections are point like and there are no known local pseudo-symmetries in the crystal or other prior constraints. In the last column we also list the estimated percentage of the total number of PDB entries for that space group.

Space group	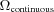 (≥)	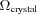 (≥)	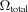 (≥)	% of PDB
Fig. 1 ▸ (tight support)	= 1.9	= 0.7	= 2.6	NA
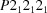	1	1/2	3/2	23.3
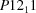	2	1/2	5/2	16.7
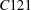	1	1/2	3/2	9.8
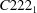	1/2	1/2	1	5.1
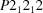	1	1/2	3/2	5.1
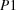	4	1/2	9/2	4.0
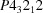	1/2	1/2	1	3.9
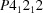	1/2	1/2	1	3.2
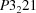	2/3	1/2	7/6	3.2
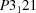	2/3	1/2	7/6	3.0
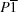	8	1	9	0.02

**Table 3:** Simulation and reconstruction parameters used in Fig. 5 ▸ 3D dimensions are given as *x*, *y*, *z* values.

Parameters	Values
*N*	100^3^
σ	0.6 Å
Diffraction grid	128, 128, 128
Real-space domain	51, 109, 158 (Å)
Space group	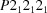
Iteration sequence	6 × (500 DM then 500 ER)
DM: β	0.8
Support update frequency	20 iterations
Support smoothing parameter	0.5 Å
Voxels (volume of rigid unit)	46658 (111 nm^3^)

## Footnotes

1 Recent work from Chapman *et al*. provides a statistical model for the continuous diffraction intensities. Under the assumptions listed above, it becomes possible to predict (for example) the number of rigid units in the crystal. Therefore, their model could in principle be adapted as a prior test for translational disorder (Chapman *et al.*, 2017 ▸).

2
https://github.com/andyofmelbourne/crappy_crystals.

3 By ‘linearly independent equations’ we mean here the number of equations relating the unknown quantities to the known quantities that are not linearly related by known constraints or symmetries of the system.

4
https://github.com/andyofmelbourne/crappy_crystals.

## Appendix A Noise model

In simulating the diffraction intensities of the disordered crystal we have included the effect of photon-counting statistics from a flat 2D detector in the far-field of the crystal. We have assumed that the diffraction intensities are obtained in a serial collection scheme, for example at a synchrotron or a free-electron laser facility, by merging many 2D diffraction images from all orientations of the crystal. The mean value of  is then equal to the total number of photons detected at this point in -space (within a given binning radius or voxel size) divided by the number of times this voxel was intersected by a detector pixel, which is proportional to the inverse of the scattering angle  (if the crystal orientations were evenly sampled). This scaling rule applies for resolution shells that fall fully within the 2D extent of the detector and does not account for detector gaps or corners. The decrease in the solid angle for pixels at higher diffraction angles has no effect on this scaling, as this simply spreads the photon counts for elements of  across more pixels. To simulate this process we therefore scaled the calculated  by , normalized this function to the total number of collected photons (which at this point represents a map of the total number of photons collected at each *q*-space bin), applied Poisson counting statistics and rescaled by *q*. In this way the calculated diffraction intensities more accurately reflected the increase in noise at higher resolution. For the simulation shown in Fig. 1 ▸ the total photon count is .

## Appendix B Estimation of the disorder length

In Fig. 6 ▸ we show the radial profile of the scattering intensities both on and off the reciprocal-lattice sites. For large crystals with sharp diffraction peaks, the scattering intensity for points off the reciprocal lattice is dominated by the continuous diffraction of the crystal, while points on the reciprocal lattice have contributions from both the first and second terms in equation (2). In both cases the average intensity in a given *q*-shell is proportional to the intensity of the computed diffraction of the rigid unit. For the reciprocal-lattice points, this is because the summation over several Bragg reflections tends to cancel the interference terms between each rigid unit in the unit cell. This is commonly assumed to be true, for example, when evaluating the so-called ‘*B* factor’ from a Wilson plot. This suggests that σ can be estimated independently of  by evaluating the ratio of the radial profiles for the on-Bragg and inter-Bragg intensities as shown in Fig. 6 ▸ (black circles). Here we make the assumption that on-Bragg intensities include contributions from both terms in equation (2) while the inter-Bragg intensities depend only on the first term. Starting from equation (2) this ratio  can be approximated by

if the radial average of the normalized reciprocal-lattice function, given by

where  lie on the reciprocal-lattice points, is known, then  is better approximated by

For our simulation, a least-squares fit of equation (25) to the ratio  (determined from the noisy diffraction data) provides a good estimate for the disorder length ( Å versus 0.6 Å). The curve fit to  is shown as the blue line in Fig. 6 ▸. Because σ is determined from the ratio of diffraction intensities at equal diffraction angles, they are invariant to other factors that might scale the radial intensity such as the *q*-dependent falloff in the scattering intensity due to the atomic form factors or uncorrelated atomic disorder in the crystal.

Note that this fitting procedure assumes that the translational disorder is isotropic, with the displacements following a normal distribution. However this is not required for the reconstruction algorithm and this fitting procedure could likely be modified to account for alternative models for the rigid-body motion.

## Appendix C Estimation of the number of rigid units

The distribution of Bragg peak intensities arising from macromolecular crystals inside a given resolution shell has long been known to follow a particular distribution, described by Wilson statistics. The continuous diffraction intensities arising from a single orientation of the rigid unit will follow this same distribution. However the distribution of the sum of diffraction intensities from different orientations of the rigid unit is equal to the convolution of the distributions of those intensities alone. The distribution of the continuous diffraction intensities in this context has been studied extensively in recent work by Chapman *et al.* (2017 ▸). There they show that a modified form of Wilson statistics can be used not only to estimate *q*-dependent background levels in individual diffraction frames, but also to identify the number of independent rigid units, that is unique types of rigid units, in the crystal as a whole.

## Appendix D Metrics

As it is the Bragg reflections that encode the rigid unit’s position relative to the crystal symmetry axes, the reconstruction from the continuous diffraction alone (which lacks this information) will be shifted with respect to its true location. To account for this, our fidelity metric has been minimized with respect to a shift in real space of the retrieved rigid unit () relative to the ground truth (), so that our fidelity metric can be written as

Also, as any one of the rigid units may be retrieved (without loss of generality),  was calculated against each of the rigid units in the unit cell and the minimum value was chosen.

Another measure of the reconstruction fidelity is the FSC (Frank, 2006 ▸), which measures the normalized cross-correlation coefficient between the ground truth and reconstructed volumes for each resolution shell in Fourier (or reciprocal) space. This is shown in Fig. 5 ▸ (bottom), where we plot the real part of

where *F* and  are one of the  (chosen above) for the ground truth and the reconstruction, respectively.

## References

[bb1] Ayyer, K. *et al.* (2016). *Nature*, **530**, 202–206. 10.1038/nature16949 PMC4839592 26863980

[bb4] Bates, R. H. T. (1982). *Optik*, **61**, 247–262.

[bb2] Bates, R. (1984). *Comput. Vis. Graph. Image Process.* **25**, 205–217.

[bb3] Bates, R. H. & McDonnell, M. J. (1986). *Image Restoration and Reconstruction*. Oxford: Clarendon Press.

[bb25] Bauschke, H. H., Combettes, P. L. & Luke, D. R. (2002). *J. Opt. Soc. Am. A*, **19**, 1334–1345. 10.1364/josaa.19.001334 12095200

[bb5] Borwein, J. M., Lindstrom, S. B., Sims, B., Schneider, A. & Skerritt, M. P. (2018). *Set-Valued and Variational Analysis*, **26**, 385–403.

[bb6] Bricogne, G. (1974). *Acta Cryst.* A**30**, 395–405.

[bb7] Bruck, Y. & Sodin, L. (1979). *Opt. Commun.* **30**, 304–308.

[bb8] Brünger, A. T. (1992). *Nature*, **355**, 472–475. 10.1038/355472a0 18481394

[bb9] Chapman, H. N., Yefanov, O. M., Ayyer, K., White, T. A., Barty, A., Morgan, A., Mariani, V., Oberthuer, D. & Pande, K. (2017). *J. Appl. Cryst.* **50**, 1084–1103. 10.1107/S160057671700749X PMC5541353 28808434

[bb10] Chen, J. P. J., Arnal, R. D., Morgan, A. J., Bean, R. J., Beyerlein, K. R., Chapman, H. N., Bones, P. J., Millane, R. P. & Kirian, R. A. (2016). *J. Opt.* **18**, 114003.

[bb11] Chruszcz, M., Potrzebowski, W., Zimmerman, M. D., Grabowski, M., Zheng, H., Lasota, P. & Minor, W. (2008). *Protein Sci.* **17**, 623–632. 10.1110/ps.073360508 PMC2271157 18359856

[bb12] Dronyak, R., Liang, K. S., Stetsko, Y. P., Lee, T. K., Feng, C. K., Tsai, J. S. & Chen, F. R. (2009). *Appl. Phys. Lett.* **95**, 2009–2011.

[bb13] Eberly, D. (2011). *Geometric Tools*, LLC. https://www.geometrictools.com/.

[bb14] Elser, V. (2003). *Acta Cryst.* A**59**, 201–209. 10.1107/s0108767303002812 12714770

[bb15] Elser, V. (2017). *J. Glob. Optim.* **68**, 329–355.

[bb16] Elser, V. & Millane, R. P. (2008). *Acta Cryst.* A**64**, 273–279. 10.1107/S0108767307050684 18285621

[bb17] Fienup, J. R. (1978). *Opt. Lett.* **3**, 27–29. 10.1364/ol.3.000027 19684685

[bb18] Fienup, J. R. (1987). *J. Opt. Soc. Am. A*, **4**, 118.

[bb19] Frank, J. (2006). *Three-Dimensional Electron Microscopy of Macromolecular Assemblies: Visualization of Biological Molecules in Their Native State*, pp. 130–131. Oxford University Press.

[bb20] Hart, J. C. (1994). *Distance to an Ellipsoid*, in *Graphics Gems* IV. New York: Academic Press.

[bb21] Hayes, M., Jae Lim & Oppenheim, A. (1980). *IEEE Trans. Acoust. Speech Signal. Process.* **28**, 672–680.

[bb22] He, H. & Su, W.-P. (2015). *Acta Cryst.* A**71**, 92–98. 10.1107/S2053273314024097 25537392

[bb23] Liu, Z.-C., Xu, R. & Dong, Y.-H. (2012). *Acta Cryst.* A**68**, 256–265. 10.1107/S0108767311053815 22338660

[bb24] Lo, V. L., Kingston, R. L. & Millane, R. P. (2016). *J. Struct. Biol.* **196**, 407–413. 10.1016/j.jsb.2016.09.004 27623229

[bb26] Marchesini, S. (2007). *Rev. Sci. Instrum.* **78**, 1–10. 10.1063/1.2403783 17503899

[bb27] Marchesini, S., He, H., Chapman, H. N., Hau-Riege, S. P., Noy, A., Howells, M. R., Weierstall, U. & Spence, J. C. H. (2003). *Phys. Rev. B*, **68**, 140101.

[bb28] Millane, R. P. & Arnal, R. D. (2015). *Acta Cryst.* A**71**, 592–598. 10.1107/S2053273315015387 26522408

[bb29] Millane, R. P. & Lo, V. L. (2013). *Acta Cryst.* A**69**, 517–527.

[bb30] Millane, R. P. & Stroud, W. J. (1997). *J. Opt. Soc. Am. A*, **14**, 568.

[bb50] Nissen, M. S., Kumar, G. N., Youn, B., Knowles, D. B., Lam, K. S., Ballinger, W. J., Knowles, N. R. & Kang, C. (2009). *Plant Cell*, **21**, 861–875. 10.1105/tpc.108.064717 PMC2671694 19304935

[bb31] Nyquist, H. (2002). *Proc. IEEE*, **90**, 280–305.

[bb32] Pettersen, E. F., Goddard, T. D., Huang, C. C., Couch, G. S., Greenblatt, D. M., Meng, E. C. & Ferrin, T. E. (2004). *J. Comput. Chem.* **25**, 1605–1612. 10.1002/jcc.20084 15264254

[bb33] RCSB (2018). PDB Data Distribution by Space Group. https://www.rcsb.org/stats/distribution_space-group.

[bb34] Rupp, B. (2009). *Biomolecular Crystallography: Principles, Practice, and Application to Structural Biology*, 1st ed. New York: Garland Science.

[bb35] Sayre, D. (1952). *Acta Cryst.* **5**, 843.

[bb36] Shannon, C. (1949). *Proc. IRE*, **37**, 10–21.

[bb37] Thibault, P. & Elser, V. (2010). *Annu. Rev. Condens. Matter Phys.* **1**, 237–255.

[bb38] Thibault, P., Elser, V., Jacobsen, C., Shapiro, D. & Sayre, D. (2006). *Acta Cryst.* A**62**, 248–261. 10.1107/S0108767306016515 16788265

[bb39] Wukovitz, S. W. & Yeates, T. O. (1995). *Nat. Struct. Biol.* **2**, 1062–1067. 10.1038/nsb1295-1062 8846217

